# Severe metabolic acidosis with renal tubular acidosis features attributed to topical brinzolamide in a patient with stage 3 chronic kidney disease: a case report

**DOI:** 10.1186/s12882-026-05167-7

**Published:** 2026-07-07

**Authors:** Ming-Yuan Victor Chao, Yi-chun Wang

**Affiliations:** 1 Department of Internal Medicine, Taipei Medical University Shuang-Ho Hospital Ministry of Health and Welfare, New Taipei City, Taiwan; 2 Division of Nephrology, Taipei Tzu Chi Hospital, Buddhist Tzu Chi Medical Foundation, Taipei, Taiwan; 3 School of Medicine, Tzu Chi University, Hualien, Taiwan

**Keywords:** Renal tubular acidosis, Carbonic anhydrase inhibitors, Brinzolamide, Metabolic acidosis, Chronic kidney disease, Urine anion gap, Glaucoma, Acute kidney injury, Naranjo scale, Case report

## Abstract

**Background:**

Metabolic acidosis is common in chronic kidney disease (CKD). However, when the severity of acidosis is disproportionate to renal dysfunction, exogenous causes must be excluded. Topical carbonic anhydrase inhibitors (CAIs) used for glaucoma are commonly perceived as locally acting; in patients with normal renal function their plasma free-drug concentrations are far below those required for systemic effects. In CKD, however, reduced clearance and reduced renal acid-excretory reserve can unmask systemic toxicity. Individual recognition of the syndrome remains uncommon at the bedside, particularly when it presents in the context of acute-on-chronic kidney injury, where its laboratory features can be obscured by acute kidney injury(AKI)-related acidosis.

**Case presentation:**

A 73-year-old Asian male with stage 3 CKD and glaucoma presented with progressive dyspnea, anorexia, and a 3-kg weight loss one month after starting fixed-combination brinzolamide 1% / timolol 0.5% eye drops. Laboratory evaluation revealed severe metabolic acidosis (pH 7.29; serum bicarbonate 8.9 mmol/L, from a pre-exposure baseline of 20.3 mmol/L) and acute-on-chronic kidney injury (creatinine 3.9 mg/dL versus baseline ~ 2.0 mg/dL). The disturbance was mixed: a delta-delta ratio of 0.42 (calculated using a reference normal serum bicarbonate of 24 mmol/L) indicated a substantial non-anion-gap (hyperchloremic) component superimposed on a smaller high-anion-gap component, with hypokalemia (K^+^ 3.3 mmol/L), hyperchloremia (Cl^-^ 107 mmol/L), positive urine anion gap (+ 33.4 mmol/L), and a fractional excretion of bicarbonate of 4.3% measured during established acidosis (without bicarbonate loading). Other causes of metabolic acidosis were systematically excluded. Causality assessment by the Naranjo Adverse Drug Reaction Probability Scale yielded a score of 7 (probable). Discontinuation of the ophthalmic solution and alkali therapy resulted in the resolution of acidosis and recovery of renal function to baseline within one week, sustained at one-year follow-up.

**Conclusions:**

Topical CAIs can undergo significant systemic absorption, bypassing first-pass metabolism, and precipitate life-threatening acidosis in patients with reduced renal reserve. Clinicians must maintain a high index of suspicion for ophthalmic medications as hidden causes of mixed acid-base disorders in patients with CKD.

## Background

Metabolic acidosis is a frequent and prognostically important complication of chronic kidney disease (CKD). However, it generally develops only when the glomerular filtration rate falls below 20–25 mL/min/1.73 m², and even at advanced stages the plasma bicarbonate is typically only mildly reduced — values below 14–15 mmol/L are unusual in CKD-related acidosis alone [1]. When the severity of acidosis is therefore disproportionate to the degree of renal dysfunction, an exogenous etiology must be actively excluded.

Carbonic anhydrase inhibitors (CAIs) are well-established causes of hyperchloremic metabolic acidosis when administered orally, and the same pharmacological mechanism is exploited topically for glaucoma in fixed or single-agent ophthalmic formulations of brinzolamide and dorzolamide. Although these formulations are widely perceived as locally acting, instilled solutions drain through the nasolacrimal duct into the highly vascularized nasopharyngeal mucosa, entering the systemic circulation and bypassing hepatic first-pass metabolism [2]. The classic pharmacokinetic study by Maren et al. demonstrated that, on standard topical dorzolamide dosing, erythrocyte concentrations rise to ~ 20–25 µM and saturate carbonic anhydrase II in red cells, but that plasma free-drug concentrations remain at approximately 0.034 µM — about 1/200 of those required to produce systemic effects from oral acetazolamide [3]. The authors concluded that topical dorzolamide produces no systemic effects in subjects with normal renal function. Critically, however, the same study showed that the drug is renally cleared (renal clearance ~ 90 mL/min in healthy individuals), so reduced renal clearance in CKD predictably prolongs systemic exposure and may shift the balance toward clinically apparent toxicity [3]. Beyond metabolic acidosis, well-characterized systemic effects of CAIs include numbness and tingling of extremities, metallic taste, depression, fatigue, malaise, weight loss, decreased libido, gastrointestinal irritation, urolithiasis, and transient myopia, with rare reports of blood dyscrasias (including aplastic anemia) [4, 5].

Topical-CAI-induced acid-base disturbance has been described before. Menon and Vernon reported indirect evidence of metabolic acidosis (lethargy, dysgeusia, and oxidative blackening of a worn silver chain) in an adult started on topical brinzolamide; no formal blood gas was obtained in that case [6]. Hoffmanová and Sánchez documented overt normal-anion-gap metabolic acidosis (HCO_3_^-^ 17.3 mmol/L) accompanied by normocytic anemia and hyperkalemia in a 46-year-old man with diabetic nephropathy and moderately impaired renal function (creatinine clearance ~ 55 mL/min/1.73 m^²^) on topical dorzolamide, with full resolution after withdrawal [7]. At the population level, Wang et al. analyzed a nationwide cohort of 2,423 Taiwanese patients with advanced (pre-dialysis stage 5) CKD with glaucoma. Among the 257 patients receiving topical CAIs, the adjusted hazard ratio for hospital admission for metabolic acidosis was 1.89 (95% CI 1.07–3.36) and the adjusted hazard ratio for progression to long-term dialysis was 1.17 (95% CI 1.01–1.37) compared with non-users [8]. In daily nephrology practice, however, individual recognition remains uncommon, particularly when the patient presents with acute-on-chronic kidney injury (AKI on CKD): the laboratory features of CAI-induced renal tubular acidosis (RTA) can be obscured by, and may even be confused with, the metabolic acidosis intrinsic to AKI.

The aim of this case report is to illustrate a critical diagnostic blind spot in medication reconciliation. We present a case in which severe mixed metabolic acidosis with prominent non-anion-gap features developed one month after initiation of a topical brinzolamide-containing ophthalmic solution in a patient with stage 3 CKD and use this case to illustrate the diagnostic reasoning required to identify a CAI contribution against the background of AKI on CKD. This case report has been prepared in compliance with the CARE (CAse REport) guidelines.

## Case presentation

### Patient information

A 73-year-old Asian man with a six-year history of stage 3 CKD (baseline serum creatinine ~ 2.0 mg/dL), with an estimated glomerular filtration rate ~ 30 mL/min/1.73 m²), chronic bronchiectasis, and bilateral primary open-angle glaucoma was referred to the nephrology outpatient clinic for evaluation of progressive dyspnea, anorexia, and weight loss of one month duration. He also reported mild diffuse abdominal discomfort and a 3-kg weight loss (from 38 kg to 35 kg) over the same period. He denied fever, cough, hemoptysis, diarrhea, vomiting, melena, or polyuria. He had no history of hypertension, diabetes mellitus, hepatic disease, autoimmune disease, ureteric or renal stones, or previous episodes of metabolic acidosis.

### Medication and exposure history

His regular medications were unchanged from the preceding year and notably did not include diuretics, NSAIDs, ACE inhibitors, angiotensin-receptor blockers, lithium, or antiretroviral agents. The single recent change was the initiation, one month before presentation, of a fixed-combination ophthalmic solution containing brinzolamide 1% and timolol 0.5% (Azarga^Ⓡ^), one drop in each eye twice daily, prescribed by the ophthalmology service for inadequate intraocular pressure control. There was no history of ammonium chloride, toluene, or laxative use, no recent gastrointestinal losses, and no acute infection. Adherence to the eye drops was confirmed by family caregiver report.

### Clinical findings

On examination, the patient appeared to be alert and oriented. Blood pressure was 136/92 mmHg. Heart rate was 84 beats/min and regular, respiratory rate was slightly elevated 20/min and temperature 36.6^°^c. Chest auscultation revealed bilateral coarse breath sounds. The abdomen was soft and non-tender, and there was no costovertebral angle tenderness or peripheral edema. Neurological evaluation was unremarkable.

### Diagnostic assessment

Arterial blood gas analysis demonstrated severe metabolic acidosis (pH of 7.29; P_a_CO_2_ 18.8 mmHg; HCO_3_^-^ 8.9 mmol/L), with mild concurrent respiratory alkalosis by Winter’s formula. Serum chemistry showed creatinine at 3.9 mg/dL (versus a stable baseline of approximately 2.0 mg/dL), urea 46 mg/dL, sodium 131 mmol/L, potassium 3.3 mmol/L, chloride 107 mmol/L, and albumin 2.7 g/dL. The serum anion gap was 15.1 mmol/L; corrected for hypoalbuminemia (adding 2.5 mmol/L per 1 g/dL fall in albumin), the corrected anion gap was 18.3 mmol/L. The delta-delta ratio (Δ corrected anion gap / Δ HCO_3_^-^, calculated using reference values of 12 mmol/L for the corrected anion gap and 24 mmol/L for the serum bicarbonate) was 0.42, indicating that a non-anion-gap (hyperchloremic) component dominated and was superimposed on a smaller high-anion-gap component. The patient’s pre-exposure baseline serum bicarbonate, obtained as venous total CO_2_ on a routine basic metabolic panel at a stable nephrology follow-up visit two months before presentation (when serum creatinine was at his stable baseline of approximately 2.0 mg/dL), was 20.3 mmol/L, demonstrating that the value of 8.9 mmol/L on presentation represented an acute decrease of 11.4 mmol/L during the exposure.

Investigations directed at alternative causes of metabolic acidosis were unrevealing. Plasma lactate, β-hydroxybutyrate, and acetone were normal; the serum osmolar gap was within normal limits, making toxic alcohol ingestion unlikely. Liver function tests, including alanine aminotransferase, aspartate aminotransferase, bilirubin, and ammonia, and thyroid function were normal. Autoimmune serology (anti-nuclear antibody, anti-dsDNA, anti-SSA, and anti-SSB, complement C3 and C4) was negative.

Electrocardiogram showed left ventricular hypertrophy. Chest and abdominal imaging reported bilateral hilar bronchiectasis but otherwise non-significant. Urine indices, obtained without prior bicarbonate or ammonium chloride loading, showed urine pH 5.5, urine sodium 72 mmol/L, urine potassium 15.4 mmol/L, urine chloride 54 mmol/L, urine anion gap + 33.4 mmol/L, and fractional excretion of bicarbonate (FeHCO_3_) 4.3%. The combination of hypokalemia, hyperchloremia, a positive urine anion gap, and a low FeHCO_3_ measured during established acidosis was interpreted as compatible with renal tubular acidosis with a distal-predominant pattern and possible partial proximal reabsorptive contribution. The full laboratory profile is presented in Table 1.

**Table 1:** Laboratory data on presentation and at one week follow-up

Parameter	Initial Presentation	Day 7 follow-up	Reference Range
Serum			
Sodium, mmol/L	131		135–145
Potassium, mmol/L	3.3	4.1	3.6–5.2
Chloride, mmol/L	107		95–105
Albumin, g/dL	2.7		3.5-5.0
Creatinine, mg/dL	3.9	1.8	0.6–1.1
BUN, mg/dL	46		6–21
Arterial blood gas			
pH	7.29	7.41	7.35–7.45
PaO_2,_ mmHg	124	108	> 80
PaCO_2_, mmHg	18.8	41.1	35.0–45.0
Bicarbonate, mmol/L	8.9	25.7	22–26
Urine			
Urine pH	5.5		5.0–8.0
Urine Sodium, mmol/L	72		N/A
Urine Potassium, mmol/L	15.4		N/A
Urine Chloride, mmol/L	54		N/A

Note: Unit conversion factors: serum urea nitrogen in mg/dL to mmol/L, $\:\times\:$0.357; creatinine in mg/dL to mmol/L, $\:\times\:$88.4

Abbreviations: BUN, Blood Urea Nitrogen; PaO_2_, Partial pressure of oxygen in arterial blood; PaCO_2_, Partial Pressure of Carbon Dioxide in Arterial Blood

### Differential diagnosis

The differential considered uremic acidosis, gastrointestinal bicarbonate loss, distal (Type 1) RTA from Sjögren’s syndrome or other autoimmune disease, proximal (Type 2) RTA, hypoaldosteronism (Type 4), toxic ingestions including ethylene glycol or methanol, and drug-induced acid-base disturbance. The pattern of the disturbance, the negative etiological work-up, the close temporal relationship to a newly initiated CA-inhibiting agent, and the rapid reversal after withdrawal made drug-induced renal tubular acidosis from systemically absorbed brinzolamide the most plausible explanation. Causality was formally assessed using the Naranjo Adverse Drug Reaction Probability Scale [9], yielding a total score of 7 (cut-offs: ≥9 definite, 5–8 probable, 1–4 possible, ≤ 0 doubtful), corresponding to a ‘probable’ adverse drug reaction (Table 2).

**Table 2:** Naranjo adverse drug reaction probability scale applied to the present case

Question	Answer	Score
1. Are there previous conclusive reports on this reaction?	Yes — prior case reports (Menon, Hoffmanová) and a population-based cohort (Wang).	+ 1
2. Did the adverse event appear after the suspected drug was administered?	Yes — onset within one month of starting brinzolamide/timolol.	+ 2
3. Did the adverse reaction improve when the drug was discontinued or a specific antagonist administered?	Yes — full normalisation within one week of withdrawal and alkali therapy.	+ 1
4. Did the adverse reaction reappear when the drug was re-administered?	Not done; rechallenge ethically inappropriate.	0
5. Are there alternative causes that could on their own have caused the reaction?	No — extensive work-up excluded alternative causes. AKI on CKD alone does not plausibly explain the severity (HCO3 − 8.9 mmol/L).	+ 2
6. Did the reaction reappear when a placebo was given?	Not done.	0
7. Was the drug detected in blood or other fluids in concentrations known to be toxic?	Not measured.	0
8. Was the reaction more severe when the dose was increased, or less severe when the dose was decreased?	Not assessed.	0
9. Did the patient have a similar reaction to the same or similar drugs in any previous exposure?	No prior CAI exposure documented.	0
10. Was the adverse event confirmed by any objective evidence?	Yes — arterial blood gas, electrolyte, and urine indices.	+ 1
**Total score**	**Probable adverse drug reaction.**	**7**

Naranjo cut-offs: ≥ 9 definite; 5–8 probable; 1–4 possible; ≤ 0 doubtful. ADR, adverse drug reaction; AKI, acute kidney injury; CAI, carbonic anhydrase inhibitor; CKD, chronic kidney disease

### Therapeutic intervention

Brinzolamide/timolol eye drops were discontinued and replaced, in consultation with the ophthalmology team, with a prostaglandin-analogue eye drop that does not contain CAI. The patient received intravenous isotonic saline with sodium bicarbonate aimed at slow correction over 24 h, followed by oral sodium bicarbonate 1300 mg three times daily. Dietary advice emphasized increased fruit and vegetable intake as adjunctive alkali therapy [10]. Potassium repletion was administered. Renal-function-adjusted dosing of his other medications was reviewed.

### Follow-up and outcomes

Within 24 h of admission, dyspnea improved and acidemia began to correct. By day 7, arterial blood gas had normalized (pH 7.41; HCO_3_^-^ 25.7 mmol/L), serum potassium was 4.1 mmol/L, and serum creatinine had fallen to 1.8 mg/dL. Oral sodium bicarbonate was tapered over four weeks. At one-year follow-up, the patient remained asymptomatic, with stable renal function (creatinine 2.0–2.2 mg/dL), normal serum bicarbonate (24 mmol/L), and adequate intraocular pressure control on the alternative ophthalmic regimen. No recurrence of acidosis occurred. Patient acceptability of the management was positive.

## Discussion

This case highlights that topical ophthalmic agents can be a potent source of systemic toxicity in CKD patients, and that the resulting acid-base disturbance can be misinterpreted as the metabolic acidosis of acute kidney injury alone. Brinzolamide is a sulfonamide derivative that inhibits carbonic anhydrase (CA) isozyme II. Although intended for local effect, the solution drains through the nasolacrimal duct into the highly vascularized nasopharyngeal mucosa, allowing direct entry into systemic circulation and bypassing hepatic first-pass metabolism [2, 3]. In patients with normal renal function, plasma free-drug concentrations after topical dorzolamide are roughly 1/200 of those required for systemic effects, and Maren et al. concluded that no systemic effect is expected in such patients [3]. In CKD, however, the renal clearance pathway that normally constrains free-drug concentration is impaired, providing a plausible pharmacokinetic explanation for the susceptibility of CKD patients to systemic toxicity from topical CAIs [3, 8].

In the renal tubule, the cytosolic enzyme carbonic anhydrase II (CA-II) is essential for acid-base transport in multiple segments. In the proximal tubule, CA-II generates the intracellular protons required for apical secretion and the bicarbonate for basolateral reabsorption [11]. In the distal nephron, specifically within alpha-intercalated cells, CA-II provides the substrate protons for the apical H^+^-ATPase pump, a critical step for urinary acidification and ammonium trapping [11, 12]. Consequently, pharmacological inhibition of CA-II can mimic features of both proximal (Type 2) and distal (Type 1) RTA. In our case, the finding of a positive urine anion gap is consistent with a functional defect in distal hydrogen ion secretion (NH_4_^+^ excretion), supporting a mixed or distal-predominant RTA phenotype driven by this mechanism [13].

The diagnostic key in this case was the stepwise interpretation of the acid-base status against the patient’s own pre-exposure baseline. The pre-exposure serum bicarbonate of 20.3 mmol/L fell acutely to 8.9 mmol/L over the month of brinzolamide/timolol exposure. A cursory review might have attributed acidosis solely to uremia (High AG). However, the Delta-Delta ratio and the positive urine anion gap indicated that the non-anion-gap (hyperchloremic) component dominated. The temporal sequence between drug initiation and clinical presentation provided strong corroboration. Symptom onset, weight loss, and biochemical deterioration developed within one month of starting brinzolamide/timolol, with no other new exposures, no new acute illness, and no change in dietary or medication context. The most decisive piece of evidence, however, was the response to de-challenge: discontinuation of the agent together with alkali repletion produced complete normalization of bicarbonate within one week, with recovery of creatinine toward baseline.

A coherent pharmacological mechanism for the natriuretic component of the syndrome is provided by the recently published ADVOR trial [14]. In its pre-specified renal-function sub-analysis, intravenous acetazolamide added to loop diuretics in patients with acute decompensated heart failure significantly increased natriuresis and diuresis, with the magnitude of the diuretic effect larger in patients with low eGFR, while overall the incidence of worsening renal function during treatment was higher with acetazolamide than with placebo (40.5% vs. 18.9%). ADVOR studied intravenous acetazolamide in volume-overloaded heart failure, which is different from chronic topical brinzolamide use in our case, and a one-to-one analogy would therefore be inappropriate. Nonetheless, ADVOR provides direct pharmacological evidence that systemically active CAI exposure can produce a measurable natriuretic effect with attendant transient deterioration of renal function in susceptible patients, consistent with the gradual 3-kg weight loss and the creatinine rise observed during the month of brinzolamide/timolol exposure in our case.

This pathophysiological pattern also clarifies the mechanism of the AKI in our patient, which occurred in the absence of overt clinical signs of hypovolemia. We interpret the AKI as prerenal in origin, arising from the combination of CAI-induced natriuresis and diuresis on the one hand, and reduced oral intake during the preceding month of anorexia and the 3-kg weight loss (approximately 7.9% of body weight) on the other. Together these produced subclinical, compensated volume depletion on top of stage 3 CKD; the absence of resting orthostatic hypotension or tachycardia at presentation is consistent with compensated rather than overt hypovolemia in an elderly patient, and the rapid response of serum creatinine to intravenous isotonic saline (1.8 mg/dL by day 7) supports this prerenal interpretation.

Two important caveats must be acknowledged. First, a positive urine anion gap is not specific to RTA in the setting of AKI, as AKI can lower urinary NH_4_^+^ excretion and produce a positive UAG without an intrinsic distal defect. Second, partial recovery of bicarbonate paralleled recovery from AKI, so the relative contributions of AKI and of a discrete drug-induced RTA cannot be quantified from the clinical course alone. Definitive separation of these mechanisms would have required post-recovery provocative testing — ammonium chloride loading or measurement of the urine-blood PCO_2_ gradient under bicarbonate loading, the latter of which has high sensitivity and specificity for H^+^-ATPase-defect distal RTA [15] — but this was not undertaken because the abnormality fully resolved off the offending agent and provocative testing in a recovered, asymptomatic patient was not deemed ethically justifiable. We acknowledge that absolute proof of causality in a single-case report — which would require deliberate drug rechallenge — is not ethically feasible after a severe adverse event such as life-threatening metabolic acidosis. The accepted methodological standard for case-report-level causality assessment is therefore the application of structured probability scales, of which the Naranjo Adverse Drug Reaction Probability Scale is the most widely used. We therefore present drug-induced RTA as the most likely dominant contributor to a mixed acid-base disturbance, with AKI on CKD acting as a permissive and amplifying factor; this position is further supported by formal causality assessment using the Naranjo Adverse Drug Reaction Probability Scale, which yielded a score of 7, corresponding to a probable adverse drug reaction (Table 2) [9].

### Interpretation of FeHCO3 in established acidosis

A FeHCO_3_ of ≥ 15–20% measured under physiological serum bicarbonate is the classical threshold for proximal RTA. In our case the value of 4.3% was measured during established acidemia (HCO_3_^−^ 8.9 mmol/L), when the filtered load of bicarbonate is markedly reduced; under such conditions, even a substantial proximal re-absorptive defect can yield a low FeHCO_3_ because the absolute amount of bicarbonate filtered is small. Formal demonstration of a proximal defect would have required a bicarbonate-loading test, which was not performed. The 4.3% value is therefore best interpreted as compatible but not diagnostic of a partial proximal reabsorptive defect superimposed on a distal acidification defect, consistent with the dual-segment role of CA-II inhibition.

### Vulnerability of CKD patients and the wider literature

While acid-base homeostasis is typically maintained in patients with normal renal function despite significant acid loads, patients with CKD are uniquely vulnerable. As detailed by Kraut and Madias, while residual nephrons in CKD adapt via increased per-nephron ammoniagenesis, the critical reduction in total nephron mass eventually limits the overall capacity [1]. Renal clearance of CAIs is also reduced, prolonging systemic exposure [3]. Consistent with this physiology, the nationwide population-based cohort by Wang et al. reported that pre-dialysis stage 5 CKD patients newly exposed to topical CAIs had an adjusted hazard ratio of 1.89 (95% CI 1.07–3.36) for hospital admission for metabolic acidosis and a significantly higher rate of progression to long-term dialysis (adjusted HR 1.17; 95% CI 1.01–1.37) compared with non-users [8]. Although the population-level signal is established, individual recognition at the bedside remains uncommon, and the present case is offered as a clinically instructive illustration of how the syndrome can present, be reasoned about, and be reversed. Interestingly, this case also highlights the utility of dietary interventions. Evidence suggests that increasing dietary alkali through fruits and vegetables can be as effective as oral sodium bicarbonate in correcting metabolic acidosis in CKD, with potential cardiovascular benefits [10].

**Table 3:** Summary of notable key points

1. Systemic Absorption Matters: Topical ophthalmic medications, such as brinzolamide and dorzolamide, can be absorbed systemically and reach systemically relevant concentrations sufficient to cause systemic toxicity, including severe metabolic acidosis.
2. CKD is a Major Risk Factor: Patients with chronic kidney disease are at high risk for CAI-induced acidosis due to impaired drug excretion and reduced capacity for acid-base compensation.
3. Diagnostic Rigor: In complex acid-base disorders, calculating the Delta-Delta ratio is essential. It helps identify mixed disorders (e.g., concomitant RTA) that might be masked by a high anion gap metabolic acidosis associated with AKI.
4. Dietary Management: In addition to pharmacological alkali therapy, increasing fruit and vegetable intake is an evidence-based strategy to manage metabolic acidosis in CKD patients, helping to preserve renal function and improve outcomes.

### Strengths and limitations

The principal strengths of this report are the systematic exclusion of alternative causes of acidosis, the application of formal causality assessment using the Naranjo scale, the extended one-year follow-up that documents sustained reversal of the acid-base disturbance after withdrawal, and the explicit recognition of the diagnostic constraints imposed by concurrent AKI. Several limitations must, however, be acknowledged. As a single-patient observation, the report cannot prove individual causality beyond temporal and pharmacological plausibility, even though a population-level association is established. Definitive distinction of drug-induced RTA from the metabolic acidosis of AKI on CKD would require post-recovery provocative testing (NH_4_Cl loading or measurement of the urine-blood PCO_2_ gradient under NaHCO_3_ loading), neither of which was undertaken in this clinically recovered patient. The FeHCO_3_ was measured during established acidosis, which limits its interpretive value as a marker of proximal RTA, and serum or urinary brinzolamide concentrations were not measured. Finally, the Wang et al. cohort enrolled patients with advanced (pre-dialysis stage 5) CKD on erythropoiesis-stimulating agents, whereas our patient had stage 3 CKD; the population-level findings therefore support biological plausibility but do not directly establish the magnitude of risk for stage 3 disease.

## Conclusion

Topical carbonic anhydrase inhibitors can precipitate severe, potentially life-threatening metabolic acidosis in patients with reduced renal reserve, even when their plasma free-drug concentrations would be subclinical in patients with normal kidney function. Although the underlying pharmacology and the population-level risk are not novel, individual recognition of the syndrome at the bedside remains suboptimal — particularly in CKD patients who present with concurrent acute kidney injury, in whom the laboratory signature of a CAI-induced renal tubular acidosis can be partially masked by AKI-related acidosis. The case presented here illustrates that disproportionate severity of acidosis, a hyperchloremic-dominant disturbance pattern and an appropriate temporal relationship to drug initiation should prompt active consideration of an exogenous CAI source, even when the agent in question is topical ophthalmic preparation. We therefore propose that ophthalmic medications be routinely captured during nephrology medication reconciliation, that acid-base status be monitored within the first 1–2 months of initiating topical CAIs in CKD patients, and that formal causality assessment be undertaken whenever an adverse drug reaction is suspected. The summary of notable key points is presented in Table 3. The Greater awareness of this under-recognized diagnostic blind spot has the potential to prevent unnecessary morbidity in a growing population of elderly patients in whom glaucoma and CKD coexist.

## Data Availability

No datasets were generated or analysed during the current study.

## Abbreviations

ACE: Angiotensin-converting enzyme
ADR: Adverse drug reaction
ADVOR: Acetazolamide in Decompensated heart failure with Volume Overload (trial)
AG: Anion gap
AKI: Acute kidney injury
BUN: Blood urea nitrogen
CA-II: Carbonic anhydrase isoenzyme II
CAIs: Carbonic anhydrase inhibitors
CI: Confidence interval
CKD: Chronic kidney disease
dsDNA: Double-stranded deoxyribonucleic acid
eGFR: Estimated glomerular filtration rate
FeHCO₃: Fractional excretion of bicarbonate
HR: Hazard ratio
NSAID: Nonsteroidal anti-inflammatory drugs
RTA: Renal tubular acidosis
SSA: Sjögren’s-syndrome-related antigen A
SSB: Sjögren’s-syndrome-related antigen B
UAG: Urine anion gap
